# METTL3/YTHDF2 m^6^A axis promotes tumorigenesis by degrading SETD7 and KLF4 mRNAs in bladder cancer

**DOI:** 10.1111/jcmm.15063

**Published:** 2020-03-03

**Authors:** Haiyun Xie, Jiangfeng Li, Yufan Ying, Huaqing Yan, Ke Jin, Xueyou Ma, Liujia He, Xin Xu, Ben Liu, Xiao Wang, Xiangyi Zheng, Liping Xie

**Affiliations:** ^1^ Department of Urology School of Medicine The First Affiliated Hospital Zhejiang University Hangzhou China

**Keywords:** bladder cancer, carcinogenesis, METTL3/YTHDF2 m^6^A axis, mRNA degradation, RNA modification

## Abstract

N6‐Methyladenosine (m^6^A) modification, the most prevalent modification of eukaryotic messenger RNA (mRNA), is involved in the progression of various tumours. However, the specific role of m^6^A in bladder cancer (BCa) is still poorly understood. In this study, we demonstrated the tumour‐promoting function and specific regulatory mechanism of m^6^A axis, consisting of the core ‘writer’ protein METTL3 and the major reader protein YTHDF2. Depletion of METTL3 impaired cancer proliferation and cancer metastasis in vitro and in vivo. Through transcriptome sequencing, m^6^A methylated RNA immunoprecipitation (MeRIP) and RIP, we determined that the METTL3/YTHDF2 m^6^A axis directly degraded the mRNAs of the tumour suppressors SETD7 and KLF4, contributing to the progression of BCa. In addition, overexpression of SETD7 and KLF4 revealed a phenotype consistent with that induced by depletion of the m^6^A axis. Thus, our findings on the METTL3/YTHDF2/SETD7/KLF4 m^6^A axis provide the insight into the underlying mechanism of carcinogenesis and highlight potential therapeutic targets for BCa.

## 1 INTRODUCTION

Bladder cancer (BCa) is the most common malignant tumour of the urinary tract and the 10th most common type of carcinoma worldwide. Approximately 549 000 new cases and 200 000 deaths were estimated by GLOBOCAN in 2018.^1^ In 2019, approximately 80 470 patients (including 61 700 men) were diagnosed with BCa and 17 670 patients (including 12 870 men) died from BCa in the United States; thus, BCa ranks the forth in incidence and eighth in mortality in men.^2^ The increasing trend in these numbers constantly urges researchers to better understand the mechanisms underlying the pathogenesis of BCa to identify potential therapies against BCa.

m^6^A, a modification first identified in mRNA‐enriched RNA fractions in 1974,^3^ refers to methylation of the N6 position of adenosine bases, which are widely distributed in the mammalian mRNA.^4^, ^5^ With the application of available methods for detecting m^6^A, insights into the regulatory mechanism have been revealed in recent years. m^6^A RNA modification is a dynamic and reversible posttranscriptional modification process maintained by a multicomponent methyltransferase ‘writer’ complex (METTL3, METTL14 and their cofactors such as WTAP, etc)^6^, ^7^, ^8^ and removed by demethylase ‘eraser’ (FTO and ALKBH5).^9^, ^10^ The function of m^6^A in mRNA metabolism primarily depends on reader proteins, which include YT521‐B homology (YTH) domain family (YTHDF1‐3, YTHDC1‐2) and IGF2 mRNA binding protein family.^11^ Recognition by YTHDF2 could induce mRNA degradation^12^ and the bindings of YTHDF1 and YTHDF3 could initiate the translation of m^6^A‐containing transcripts^13^, ^14^ while identification by IGF2BP family could promote the stability of target mRNA.^15^

Studies documenting the importance of m^6^A modification in physiological bioprocesses and the pathogenesis of diseases have emerged in large numbers, mainly involving the regulation of cell fate decisions, embryonic development, and different kinds of cancers.^11^, ^16^ For example, knockout of m^6^A catalysing enzyme METTL3 is embryonic lethal during mouse development due to the failed termination of murine naïve pluripotency.^17^ Conditional knocking out the m^6^A reader protein YTHDF2 in mice, delayed degradation of neuron differentiation‐related m^6^A‐containing mRNAs impaired the self‐renewal of neural stem/progenitor cells and the spatiotemporal generation of neurons leading to neural development deficiency.^18^ Moreover, the overexpression of FTO in acute myeloid leukaemia could prevent the cell differentiation but promote oncogene‐mediated hematopoietic cell transformation and enhances leukemogenesis.^19^ However, the mechanism of tumorigenesis influenced by m^6^A modification dysregulation in BCa remains elusive.

In this study, we explored the biological role of m^6^A modification in BCa and principally focused on the regulatory mechanism of the m^6^A axis mediated by METTL3 and YTHDF2. We demonstrated that the m^6^A/SETD7/KLF4 axis was involved in the progression of BCa. Consequently, we hope that this study will offer an insight into the underlying molecular mechanism of tumorigenesis and provide potential therapeutic targets for BCa.

## 2 MATERIALS AND METHODS

### 2.1 Cell culture and transfection

The human BCa cell lines T24, UM‐UC‐3 and the normal human urothelium cell line SV‐HUC‐1 were obtained from the Cell Bank of Type Culture Collection of the Chinese Academy of Sciences and verified by short tandem repeat DNA profiling analysis. SV‐HUC‐1 and T24 cell lines were cultured in RPMI 1640 medium (Corning); UM‐UC‐3 cell line was cultured in Minimum Essential Medium (MEM, Corning) supplemented with 10% heat‐inactivated foetal bovine serum (FBS, BI) at 37°C and 5% CO_2_ in humid environment. Plasmids were transfected into cells by FuGene HD transfection reagent (Promega), and siRNAs were transfected with Lipofectamine 2000 (Invitrogen) according to the manufacturer's instructions.

### 2.2 Plasmids and small interfering RNAs

The overexpression plasmids pYTHDF2, pSETD7 and control pNull were commercially obtained from GeneChem Company. And all small interfering RNAs (siRNAs) were chemically synthesized by GenePharma Company. All corresponding sequences are summarized: negative control (NC): 5′‐ACUACUGAGUGACAGUAGA‐3′; siMETTL3‐1: 5′‐CCUGCAAGUAUGUUCACUATT‐3′; siMETTL3‐2: 5′‐GCUACCUGGACGUCAGUAUTT‐3′; siMETTL3‐3: 5′‐GGUUGGUGUCAAAGGAAAUTT‐3′; siYTHDF2‐1: 5′‐GCUCUGGAUAUAGUAGCAATT‐3′; siYTHDF2‐2: 5′‐GCGGGUCCAUUACUAGUAATT‐3′; siSETD7‐1: 5′‐GAACUUUGUUCACGGAGAATT‐3′; siSETD7‐2: 5′‐CAGUGUACCACUUUGAUAATT‐3′; siSETD7‐3: 5′‐GCAUCUACGAUAUGUUUGUTT‐3′. To avoid off‐target effects, different siRNAs of genes were merged together as the siRNA pool to cotransfect BCa cell lines in all interference experiments.

### 2.3 Lentiviruses and infection

Lentivirus‐based short‐hairpin RNA (shRNA) was used to achieve stable depletion of target genes. The vector used in this study was GV344 vector (hU6‐MCS‐Ubiquitin‐firefly_Luciferase‐IRES‐puromycin; GeneChem) which can produce luciferase for in vivo imaging. The lentivirus infection was manipulated referring to instructions. And the corresponding shRNAs are listed: shNC: 5′‐TTCTCCGAACGTGTCACGT‐3′; shMETTL3‐1: 5′‐gcAAGTATGTTCACTATGAAA‐3′; shMETTL3‐2: 5′‐cgTCAGTATCTTGGGCAAGTT‐3′.

### 2.4 Immunohistochemistry

Immunohistochemistry (IHC) analysis was performed in xenograft tumour tissues obtained from mice. The specific manipulation was described as previous study.^20^ The associated antibodies used is listed as follows: anti‐METTL3 (#ab195352, Abcam); anti‐SETD7 (#ab14820, Abcam); and anti‐KLF4 (#12173S, Cell Signaling Technology).

### 2.5 Western blot assay

Total proteins were extracted by lysing cells in 6‐well plate with 60 μL RIPA buffer supplemented with protease inhibitors (#G2006, Wuhan Goodbio Technology) and phosphatase inhibitors (#G2007, Wuhan Goodbio Technology) and quantified by BCA protein assay kit (#P0011, Beyotime). One microgram of proteins was separated by 10% SDS‐PAGE and transferred to PVDF membrane (#IPVH00010, Merck Millipore). The membrane was blocked with 5% Non‐Fat Milk (#A600669, Sango Biotech) for 1 hour at room temperature and incubated with specific antibody at 4°C overnight followed by HRP‐conjugated secondary antibody incubation for 1 hour at room temperature. Images of membrane with proteins were taken with imager (Bio‐rad ChemiDoc MP, Bio‐rad). The antibodies are recorded: anti‐GAPDH (#60004‐1‐lg, Proteintech), anti‐YTHDF2 (#24744‐1‐AP, Proteintech), anti‐E‐cadherin (#20874‐1‐AP, Proteintech), anti‐N‐cadherin (#66219‐1‐AP, Proteintech), anti‐VIMENTIN (#10366‐1‐AP, Proteintech), anti‐MMP9 (#10375‐2‐AP, Proteintech), anti‐MMP2 (#10373‐2‐AP, Proteintech), anti‐SNAIL (#13099‐1‐AP, Proteintech), anti‐CDK6 (#14052‐1‐AP, Proteintech), anti‐CCND1 (#26939‐1‐AP, Proteintech), anti‐CDK4 (#11026‐1‐AP, Proteintech), anti‐KLF4 (#12173S, Cell Signaling Technology), anti‐SLUG (#C1967, Cell Signaling Technology), anti‐METTL3 (#ab195352, Abcam) and anti‐SETD7 (#ab14820, Abcam).

### 2.6 RNA isolation and RT‐qPCR

Total RNAs were extracted with RNAiso plus (#9109, Takara). Reverse transcription was proceeded with PrimeScript RT reagent Kit (#RR036A, Takara), and quantitative PCR (qPCR) was performed by SYBR Premix Ex Taq (#RR820A, Takara) in thermocycler (CFX96 Touch Real‐Time PCR, Bio‐rad). GAPDH was chosen as the endogenous reference for calculating the relative expression of target genes with $2^{-\Delta \Delta C_{t}}$ (delta‐delta‐*C*_t_ algorithm) method. All primers are listed: SETD7 F 5′‐ATGGATAGCGACGACGAGATG‐3′; SETD7 R 5′‐GCAGAACCCGTGCGGTAAT‐3′; KLF4 F: 5′‐ACCCACACTTGTGATTACGC‐3′; KLF4 R 5′‐CCGTGTGTTTACGGTAGTGC‐3′.

### 2.7 Cell viability assay

When the confluence was up to 30%‐50% per well in 96‐wells plates, cells were transfected with merged RNA duplexes at different concentration as 0, 25, 50, 75 and 100 nmol/L. After 48 and 72 hours treatment, cell viability was detected by cell counting kit 8 test (#CK04, Dojindo) according to the protocol.

### 2.8 Colony formation assay

Five hundred treated cells (transfected with siRNAs or plasmids for 48 hours) per well were counted and seeded in 6‐well plates. After cultured for 7‐14 days, cells were fixed with methanol and then stained with 0.3% crystal violet.

### 2.9 Trans‐well assay

Nearly 5 × 10^4^ transfected cells were seeded in the trans‐well chamber (#MCEP24H48, Merck Millipore) with 0.2 mL serum‐free medium and the compartment between chamber and 24‐plate well was filled with 0.6 mL medium with 10% FBS. After culturing for 24 hours, the migrated cells were fixed on the lower surface of the membrane with methanol and stained with 0.3% crystal violet. Images were taken with phase‐contrast microscopy (IX71, Olympus) with a 20 × objective lens.

### 2.10 Wound healing assay

When treated cells grew to 100% confluence, the pipette tip (200 μL) was used to scratch the cell layer on plate to create a break. When the cellular proliferation obstructed by serum‐free medium, the cellular migration process was monitored by phase‐contrast microscope (IX71, Olympus) with 5 × objective lens.

### 2.11 Cell cycle analysis

Transfected cells were fixed in 75% ethanol overnight at 4°C. DNA staining was performed using cell cycle staining Kit (#CCS012, Multi Sciences). After 30 minutes, cells were analysed by flow cytometry (the BD LSRII Flow Cytometer System, BD Biosciences) and the data were analysed by ModFit LT 3.2 software (Verity Software House).

### 2.12 RNA m^6^A dot blot assay

RNA was isolated from cells or tissues using RNAiso plus (#9109, Takara), and the concentration was adjusted to 50 ng/μL in 36 μL RNase‐free water. Secondary structure was removed using RNA incubation buffer (mixture of MOPS, formamide and formaldehyde). Mixed up with ice‐cold 20 × Saline‐Sodium Citrate (SSC) solution (#S6639‐1L, Sigma‐Aldrich), 200 ng of RNA samples were loaded on wetted N+ membrane (#RPN303B, GE health) in dot blot apparatus. Successively, the membrane was UV cross‐linked and stained by 0.02% methylene blue (#M9140‐25G, Sigma‐Aldrich) to ensure the equal amount of total RNA by scanning. After blocked and incubated with m^6^A antibody (1:2000, #202003, Synaptic Systems) then secondary antibody, the membrane was exposed with imager (Bio‐rad ChemiDoc MP, Bio‐rad).

### 2.13 m^6^A‐RNA immunoprecipitation assay

Total RNA was extracted from two 15‐cm plates of cell lines infected by lentiviruses with the shRNA using RNAiso plus (#9109, Takara), and the process was completed according to the instructions of Magna methylated RNA immune‐precipitation (MeRIP) m^6^A Kit (#17–10499, Merck Millipore). In brief, RNA was diluted to 1 μg/μL and sheared into ~100 nt in length. After precipitation at −80°C overnight, nearly 300 μg (except 10% of input) of RNA in 300 μL of RNase‐free water was incubated with magnetic beads loaded with 10 μg of antibody of m^6^A (#ABE572, Merck Millipore) or immunoglobulin G (IgG) for 2 hours at 4°C. Then, the fragmented RNA containing m^6^A was eluted and purified with RNA purification kit (#74204, QIAGEN). Enriched fragments were analysed by RT‐qPCR. The primers were designed from the 100 nt around the predicted m^6^A peak (http://www.cuilab.cn/sramp) and listed: SETD7 F 5′‐CTGGCTTTGGGGTTCAGAGA‐3′; SETD7 R 5′‐GTCCCATTGTCAGATAAACGTAGTG‐3′; KLF4 F 5′‐CTGTGACTGGATCTTCTATCATTCC‐3′; KLF4 R 5′‐CAGTCACCCCCTTGGCATTT‐3′.

### 2.14 RNA‐binding protein immunoprecipitation assay

For RIP assay, we primarily used Magna RIP Kit (#17‐700, Merck Millipore) according to the instructions. Cells cultured in two 15‐cm plates were lysed, precipitate removed and incubated with magnetic beads loaded with 7.5 μg of antibody of YTHDF2 (#24744‐1‐AP, Proteintech) or IgG overnight at 4°C. And RNA was isolated from binding protein by proteinase K and purified followed by analysis by RT‐qPCR.

### 2.15 RNA sequencing

Total RNA was extracted from cells using RNAiso plus (#9109, Takara). The purity of RNA was assessed using the ND‐1000 NanoDrop, and the integrity was evaluated using the Agilent 2200 TapeStation (Agilent Technologies). In brief, rRNAs were removed by Epicentre Ribo‐Zero rRNA Removal Kit (Illumina). The remains were fragmented to approximately 200 bp, purified and subjected to first strand and second strand cDNA synthesis following by adaptor ligation and enrichment with a low‐cycle according to instructions of NEBNext^®^ Ultra™ RNA Library Prep Kit for Illumina (NEB). The purified I library products were evaluated using the Agilent 2200 TapeStation and Qubit^®^ 2.0 (Life Technologies) and then diluted to 10 pmol/L for cluster generation in situ on the pair‐end flow cell followed by sequencing (2 × 150 bp) HiSeq3000. After removal of reads containing adapter, ploy‐N and at low quality from raw data, the clean reads were obtained. The software HISAT2 was used to align the clean reads with default parameters, and HTSeq was employed to convert aligned short reads into read counts for each gene model. Differential expression was analysed by DEseq. The Benjamini‐Hochberg multiple test correction method was enabled and the chosen criteria of fold change >2 and adjusted *P*‐value < .05 was obeyed. The differentially expressed genes were ranked by *P*‐value, and top 200 were used for heat map in website: (http://www.ehbio.com/ImageGP/index.php/Home/Index/index.html). For KEGG enrichment analysis, a *P*‐value < .05 was used to determine significant enrichment of the gene sets.

### 2.16 Animal experiments

Male BALB/c nude mice (4 weeks old) were used in this study. For the subcutaneous implantation model, UM‐UC‐3 cells (2 × 10^6^ cells per mouse) stably METTL3 knocked down (shMETTL3‐1, shMETTL3‐2) were injected into the flanks of mice. Tumour volume was recorded every 5 days by a caliper and calculated by the formula: V = (width^2^ × length × 0.52). For tumour metastasis model, the 2 × 10^6^ UM‐UC‐3 cells (shNC, shMETTL3‐1 and shMETTL3‐2) were injected via the caudal vein per mouse. The IVIS Spectrum animal imaging system (PerkinElmer) was used to detect the tumour growth and monitor the whole metastasis in above two models with 100 μL XenoLight D‐luciferin Potassium Salt (15 mg/mL, Perkin Elmer) per mouse. Finally, mice were anesthetized and then killed for tumours and metastases which were sent for further IHC staining and lymph nodes haematoxylin‐eosin (H&E) staining. All animals were manipulated according to institutional guidelines and the permission granted by the First Affiliated Hospital, School of Medicine, Zhejiang University.

### 2.17 Statistical analysis

Statistics in this study are described as the mean ± SD in GraphPad prism. Statistical distinction between two groups was estimated by a two‐tailed Student's *t* test. Statistical significance was defined as *P*‐value of <.05.

## 3 RESULTS

### 3.1 METTL3 and YTHDF2 are highly expressed in BCa

**Figure 1:**
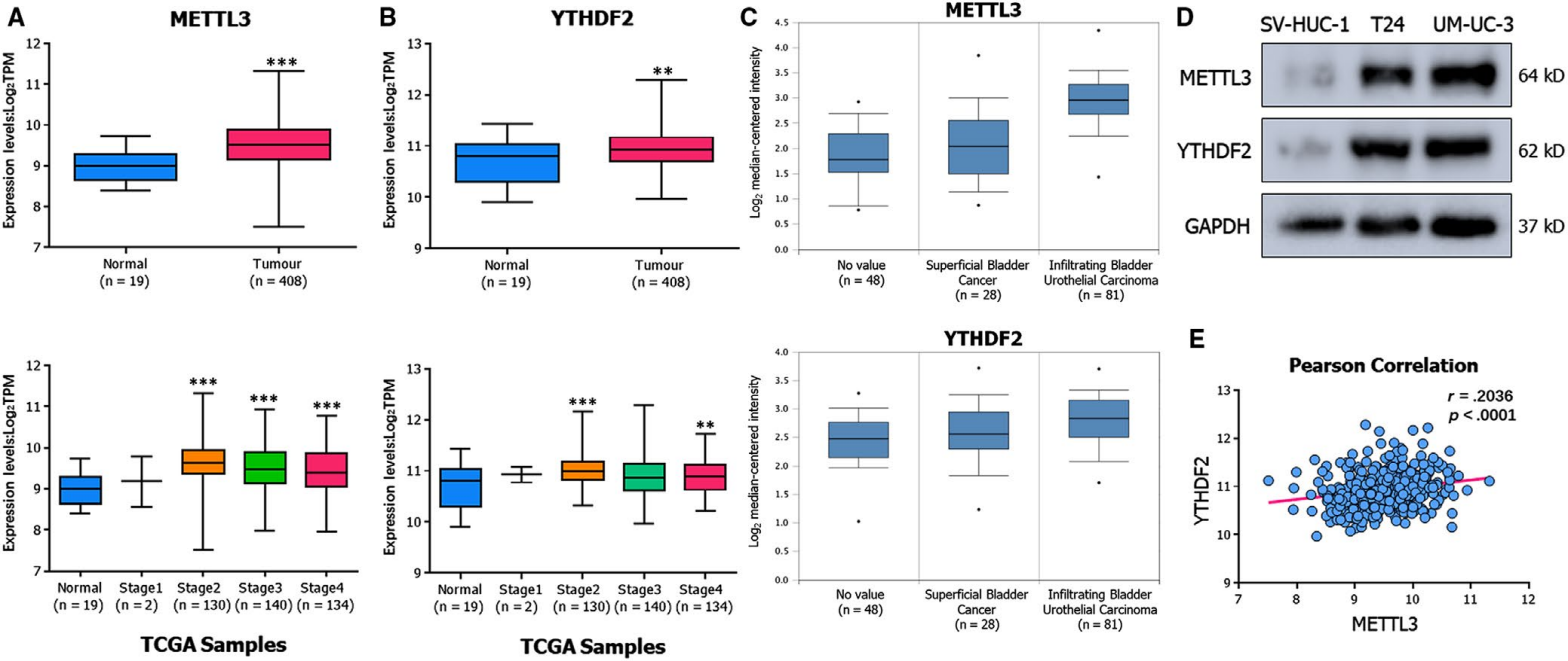
METTL3 and YTHDF2 are highly expressed in BCa. A, B, Transcript levels of METTL3 and YTHDF2 in primary tumours vs normal subjects and mRNA expression levels among stages from TCGA database. C, Expression levels of METTL3 and YTHDF2 in superficial and infiltrating BCa from Oncomine online database. D, Western blot assay showing the up‐regulated expression levels of METTL3 and YTHDF2 in BCa cancer cell lines (T24, UM‐UC‐3) compared to the human normal urothelium cell line (SV‐HUC‐1). GAPDH was used for the normalization control. E, Pearson correlation analysis of METTL3 and YTHDF2 in primary tumours (n = 408) from TCGA database; *r* = .2036, *P* < .0001. Error bars represent SD obtained from three independent experiments; *P < .05, **P < .01 and ***P < .001

To investigate the expression pattern of METTL3 and YTHDF2, data sets in the TCGA database and Oncomine online database^21^ (https://www.oncomine.org) were analysed. We observed that the transcripts of METTL3 and YTHDF2 were promoted in primary tumours compared to normal subjects (Figure 1A,B). In addition, at different cancer stages, the expression differed and displayed an increasing‐tendency as disease malignancy increased (Figure 1C). We conducted a Western blot assay to detect protein levels in distinct tumour cell lines. In contrast to the normal human urothelium cell line SV‐HUC‐1, the expression of METTL3 and YTHDF2 was indeed elevated in BCa cell lines T24 and UM‐UC‐3 (Figure 1D), consistent with the analysis of the database. As an increasing number of studies reported the crucial role of the m^6^A axis conducted by ‘writer’ and reader,^22^, ^23^ we performed the correlation analysis between ‘writer’ proteins and reader proteins (Figure S2B,C). Further correlation analysis also indicated a positive correlation between the expression of METTL3 and YTHDF2 (Figure 1E). However, the depletion of METTL3 did not directly affect the expression level of YTHDF2 (Figure S2A). Thus, we speculated that METTL3 cooperated with YTHDF2 and the METTL3/YTHDF2 m^6^A axis was involved in BCa.

### 3.2 Depletion of METTL3 impairs the proliferation and migration of BCa cells

**Figure 2:**
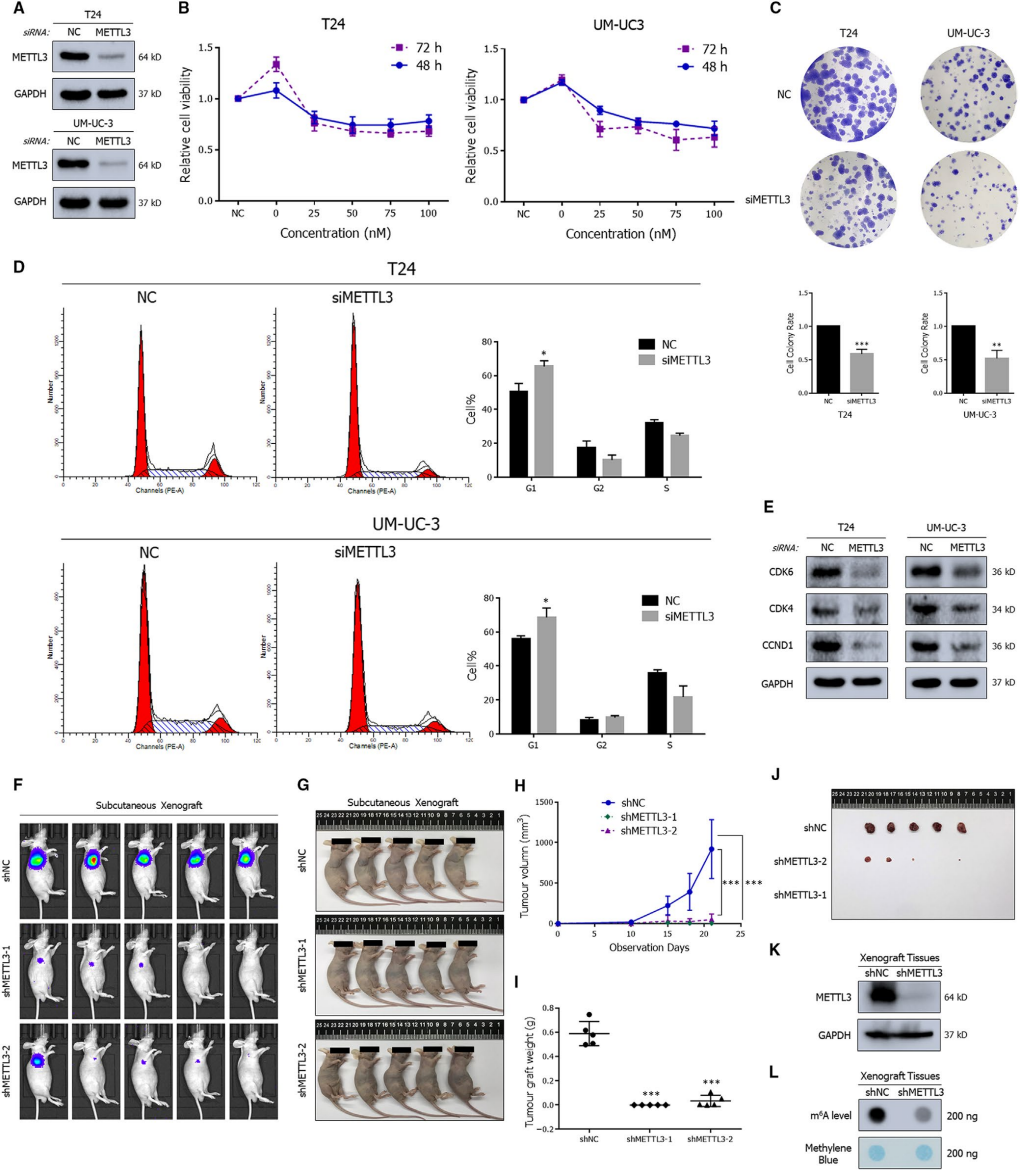
Depleted METTL3 significantly impairs proliferation of BCa. A, Western blot assay demonstrating the repressed expression of METTL3 by the siRNA pool in BCa cell lines. B, CCK‐8 assay demonstrating the decreased cell viability upon transfection with increasing concentrations of siRNA pools in cell lines. C, Colony formation assay indicates lacked METTL3 impaired colony formation ability. D, Representative cell cycle analysis demonstrating the increased percentage of cells arrested in G1 phase in METTL3‐depleted cells. E, Representative Western blots showing the altered expression of cell cycle associated proteins upon the depletion of METTL3. F‐J, Tumour subcutaneous xenograft model showing the repressed growth of xenograft tumours in tumour volumes and growth curves. K, Western blot assay confirming the knock‐down of METTL3 in UM‐UC‐3 derived xenograft tumours. GAPDH served as the normalization control. L, Dot blot assay describing the decreased m^6^A level in UM‐UC‐3 derived xenograft tumours. Error bars represent SD obtained from three independent experiments; *P < .05, **P < .01 and ***P < .001

To exclude off‐target effects, we depleted METTL3 by using a homolog‐specific siRNA pool (Figure 2A) and rescue experiments with siRNA‐resistant exogenous METTL3 were conducted (Figure S3B). We performed CCK‐8 (Figure 2B) and colony formation assays (Figure 2C) to assess cell viability in the METTL3 depleted group. CCK‐8 assay revealed marked inhibition at two time‐points and different siRNA transfection concentrations, consistent with the stunted proliferation observed in the colony formation assay. The results of cell cycle analysis indicated that the depletion of METTL3 led to cell proliferation inhibition, which was probably attributed to G1 phase cell cycle arrest (Figure 2D). The results were further confirmed by examining the expression of cell cycle related proteins including CDK4/CDK6/CCND1 (Figure 2E), which were decreased in response to depletion of METTL3. To investigate the tumour proliferation in vivo, the UM‐UC‐3 cells with stable interference of METTL3 (shMETTL3‐1 or shMETTL3‐2) and the control (shNC) were established with lentivirus‐based shRNA technique. A total of 2 × 10^6^ cells were injected into subcutaneous flank of BALB/c nude mice (4 weeks old). The results showed distinct differences between the groups in terms of luciferase activity and tumour size (Figure 2F‐L). Compared to the group infected with the non‐targeting shRNA control (shNC) lentiviruses, the knock‐down groups (shMETTL3‐1, shMETTL3‐2) had decreased luciferase activity tumour volume and m^6^A level.

**Figure 3:**
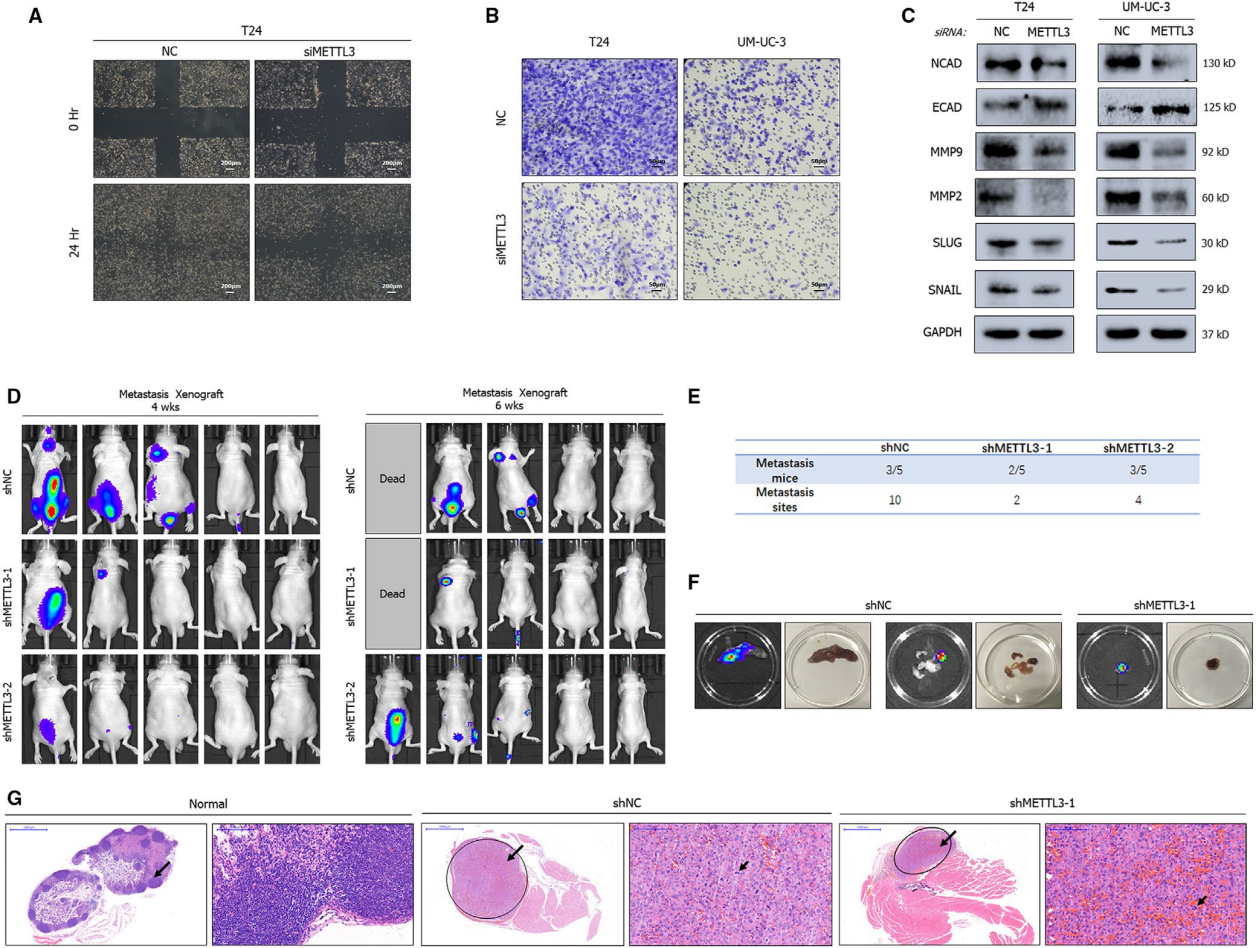
Depletion of METTL3 suppresses metastasis of BCa. A, B, Wound healing assay (A) and trans‐well assay (B) showing the inhibition of migration in METTL3‐depleted cell lines. C, Representative Western blots indicate the altered expression of EMT‐associated proteins upon METTL3 depletion by siRNA pools in cell lines. GAPDH served as the normalization control. D, E, Experimental metastases model by caudal vein injection showing the decreased metastatic ability of tumours in fluorescence intensity and the amount of metastasis sites. F, Posterior limb metastasis and lymph node metastases obtained from metastases model. G, HE staining of lymph node metastases vs normal lymph node. Smaller arrows point to tumour cells with pathologic mitosis

In addition, down‐regulation of METTL3 markedly weakened the migratory capabilities of BCa cell lines, which was confirmed by a wound healing assay (Figure 3A) and trans‐well assay (Figure 3B). Further Western blot analysis revealed the inhibition of EMT progression and the matrix metalloproteinase family (Figure 3C). To test the tumour metastasis‐promoting effect of METTL3 in vivo, we established a mouse model of experimental metastases by caudal vein injection of 2 × 10^6^ UM‐UC‐3 cells infected with lentiviruses. After more than 1 month of observation, the fluorescence intensity and the number of metastatic sites decreased in the groups shMETTL3‐1 and shMETTL3‐2 groups compared to the shNC group, suggesting significant suppression of metastasis (Figure 3D‐F). In the HE staining results, the lymph node metastases obtained from two groups were distinct from the normal one. However, the number of sites of pathological mitosis was lower in the group with stable knock‐down of METTL3 (shMETTL3‐1), showing a lower degree of malignancy (Figure 3G). In conclusion, METTL3 plays a significant role in promoting the tumorigenesis and metastasis of BCa.

### 3.3 YTHDF2 promotes the migration of BCa cancer cells

**Figure 4:**
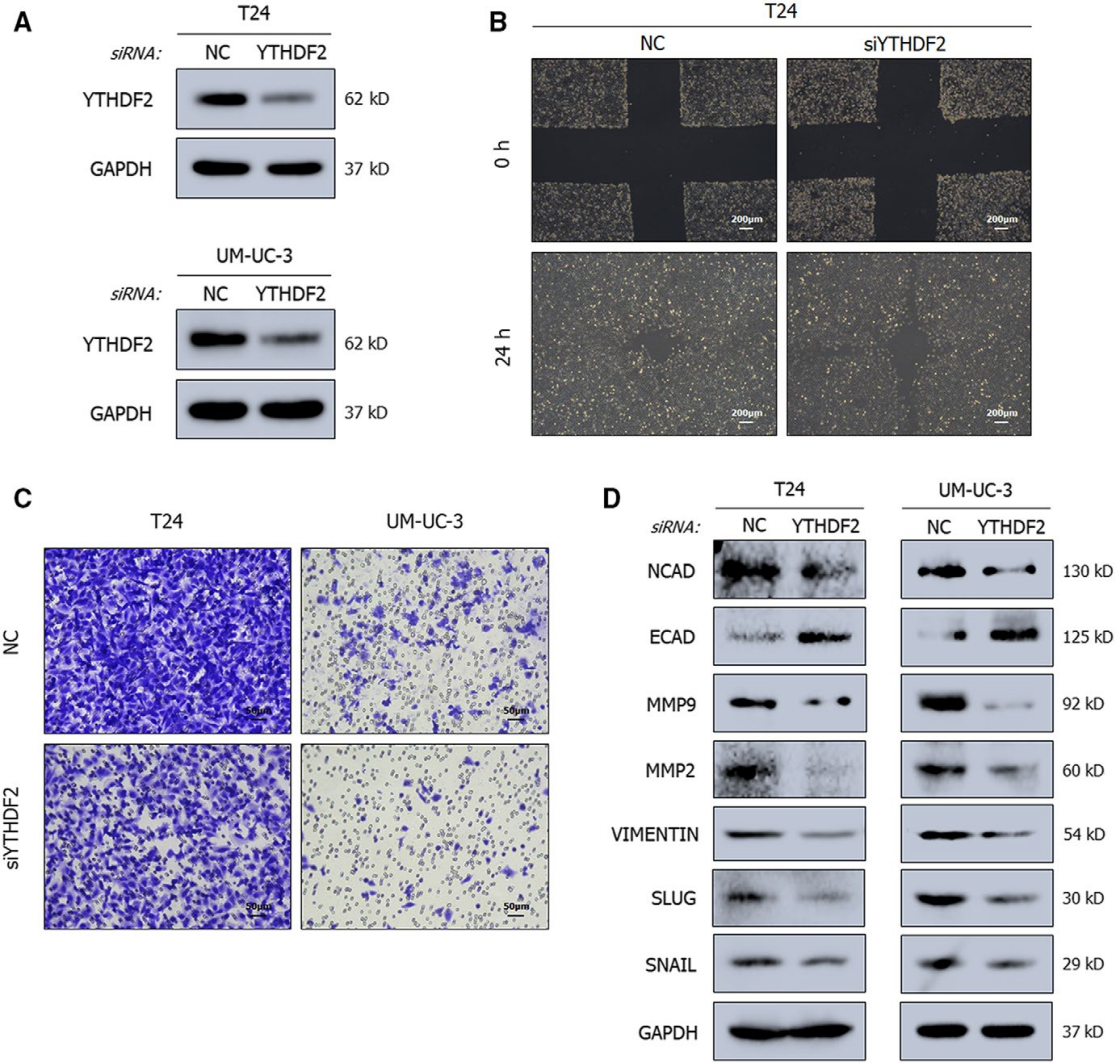
YTHDF2 promotes the migration of cancer cells. A, Representative Western blots show the suppressed expression of YTHDF2 by the siRNA pool in BCa cell lines. GAPDH served as the normalization control. B, C, Wound healing assay (B) and trans‐well assay (C) demonstrating the inhibited migration ability upon YTHDF2 depletion in cancer cell lines. D, Representative Western blots showing the altered expression level of EMT‐associated proteins. GAPDH served as the normalization control

An increasing number of studies have illustrated the importance of the m^6^A reader protein YTHDF2 in the regulation of physiological processes and diseases.^18^, ^20^ In the analysis of the TCGA database and detection in expression levels compared to SV‐HUC‐1 cells, YTHDF2 was predicted to be as a tumour promoter in BCa. To verify this, YTHDF2 was depleted in T24 and UM‐UC‐3 cells by using a homolog‐specific siRNA pool (Figure 4A). To exclude off‐target effects, rescue experiments with siRNA‐resistant exogenous YTHDF2 were conducted (Figure S3C). The depletion led to marked inhibition of cells migration, as shown by the wound healing (Figure 4B) and trans‐well assays (Figure 4C). In expression levels, a robust reduction in the migration rate and the expression levels of EMT pathway‐related proteins and matrix metalloproteinase family protein was observed after silencing YTHDF2 (Figure 4D). Conversely and concordantly, overexpression of YTHDF2 by plasmids promoted cell migration (Figure S1A‐D).

The observation of similar effects after manipulating METTL3 and YTHDF2 expression supports the concept that the axis consisting of m^6^A writer and m^6^A reader is likely to exist in BCa, analogous to a previous study in hepatocellular carcinoma.^23^

### 3.4 METTL3/YTHDF2 axis directly degrades SETD7 and KLF4 mRNAs in an m^6^A dependent manner

**Figure 5:**
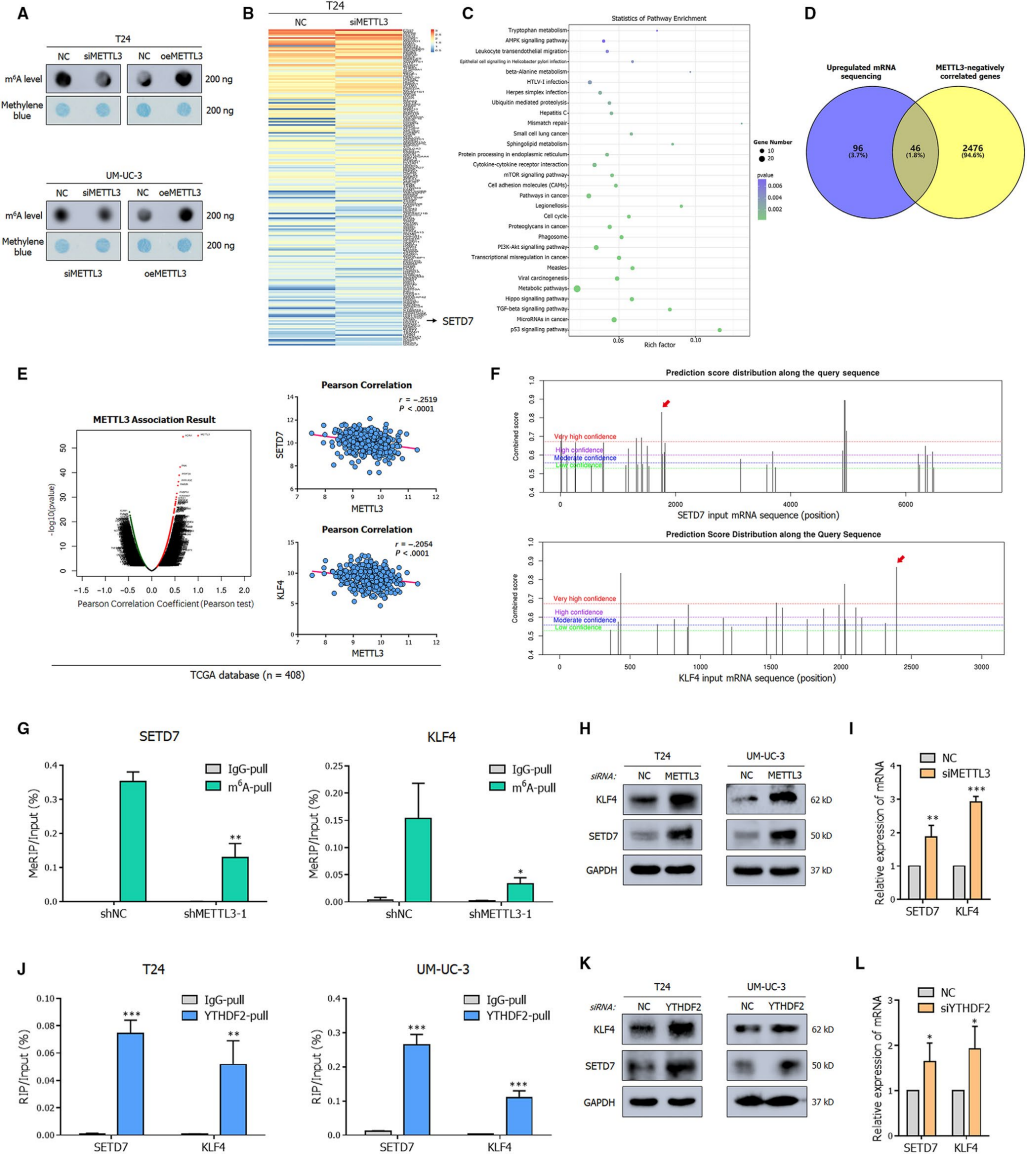
METTL3/YTHDF2 m^6^A axis directly degrades the SETD7 and KLF4 mRNAs. A, Dot blot assay demonstrates that the m^6^A level alters with changes in the expression of METTL3. B, Heat map shows representative differentially expressed genes upon METTL3‐depletion from RNA‐seq. Low level is marked blue (|log_2_FC| > 1 and *P*‐value < .05, arranging from smallest). C, KEGG enrichment analysis of differentially expressed genes upon METTL3 depletion from RNA‐seq. D, The overlap of mRNAs significantly up‐regulated (log_2_FC > 1 and *P*‐value < .05) in T24 cells upon the depletion of METTL3 and genes negatively correlated with METTL3 in TCGA database analysed by LinkedOmics is shown by Venn diagram. E, Gene correlated with METTL3 in LinkedOmics database and negative correlation of METTL3 and SETD7 (*r* = −.2519, *P* < .0001) or KLF4 (*r* = −.2054, *P* < .0001) in primary BCa from TCGA database by Pearson correlation. F, Potential m^6^A modification sites along mRNA of SETD7 and KLF4 predicted by SRAMP. The peak noted with red arrow was valid in MeRIP assay. G, *MeRIP* assay shows the decreased amount of two target mRNAs collected by m^6^A antibody upon the repressed expression of METTL3, suggesting the direct methylation of METTL3. H, Representative Western blots demonstrating up‐regulation of SETD7 and KLF4 protein upon METTL3 depletion by siRNA pools in cancer cell lines. I, The quantification of SETD7 and KLF4 mRNA abundance in METTL3 depleted T24 cells by RT‐qPCR indicates that the down‐regulation of METTL3 induced the up‐regulated expression of SETD7 and KLF4. GAPDH was used as the normalization. J, RIP assay showing the binding between YTHDF2 and SETD7 or KLF4. K, Representative Western blots documenting the accumulated expression of SETD7 and KLF4 upon down‐regulated YTHDF2 by siRNA pools in cancer cell lines. L, The quantification of SETD7 and KLF4 mRNA abundance upon depleted YTHDF2 in T24 cells by RT‐qPCR indicating the negative correlation between YTHDF2 and two targets. GAPDH was used as the normalization. Statistical significance was determined by Student's *t* test: *P < .05, **P < .01, ***P < .001

Recently, substantial studies have demonstrated the main catalytic core of METTL3 in producing m^6^A modification^6^; we obtained evidence of this catalysis in BCa by using an m^6^A dot blot assay (Figure 5A). The results indicated that overexpression of METTL3 induced a robust total m^6^A levels. To further screen the molecular targets of METTL3 and YTHDF2, transcriptome sequencing (mRNA‐seq) was performed in METTL3‐depleted T24 cells. Differentially expressed genes were selected and ranked by *P*‐value (Figure 5B); these genes were primarily enriched in AMPK signalling pathway, cell adhesion molecules (CAMs), pathways in cancer, cell cycle, PI3K‐AKT signalling pathway and transcriptional dysregulation in cancer (Figure 5C). Among the 142 up‐regulated genes identified in METTL3‐depleted T24 cells (log_2_FC > 1 and *P*‐value < .05), 46 genes were predicted to be negatively correlated with METTL3 in the TCGA database (Figure 5D). Integrating the results of the database LinkedOmics (http://www.linkedomics.org), the specific function induced by METTL3 and previous studies of our research group,^24^ SET domain containing 7 (SETD7, a histone lysine methyltransferase) and Kruppel‐like factor 4 (KLF4, an accepted transcription factor) were selected as the principal potent downstream targets (Figure 5E).

As predicted by the SRAMP database (http://www.cuilab.cn/sramp), there were at least three highly probable m^6^A‐modification peaks in these two targets (Figure 5F). To confirm the mutual interaction between METTL3 and the two downstream targets, MeRIP was performed. In contrast to the control groups, a significant decrease of mRNA enrichment by m^6^A‐specific antibody was detected in the METTL3 stably interfered UM‐UC‐3 cell lines (Figure 5G). These results confirmed that the m^6^A‐modification on target mRNAs was transferred by METTL3, and this finding was supported by Western blot and RT‐qPCR, in which depleted METTL3 enhanced the expression of SETD7 and KLF4 (Figure 5H,I). As expected, in rescue experiments, silencing SETD7 or KLF4 abrogated the tumour‐suppressive phenotypes by depletion of METTL3 (Figure S4A,B). The accumulated expression of SETD7 and KLF4 upon METTL3 depletion was also discovered in UM‐UC‐3 derived xenograft tumours obtained from nude mice (Figure S1E).

Massive studies have confirmed that METTL3 could cooperate with YTHDF2 not only in regulating cell differentiation^25^ but also causing tumour suppressor gene mRNA decay to impact the development and progression of tumour.^23^ Notably, as expected, compared with IgG pull‐down control, the methylated SETD7 and KLF4 mRNAs were abundant by the YTHDF2‐specific antibody in the RIP assay (Figure 5J). The recognition and direct binding of YTHDF2 were also demonstrated at the protein and RNA levels (Figure 5K,L). And in rescue experiments, knock‐down of SETD7 or KLF4 rescued repressed migration ability by depletion of YTHDF2 (Figure S4C,D). In summary, all these findings indicated that METTL3‐dependent m^6^A‐modification depressed the expression of SETD7 and KLF4 via YTHDF2‐mediated mRNA degradation.

### 3.5 SETD7 and KLF4 act as tumour suppressors in BCa

**Figure 6:**
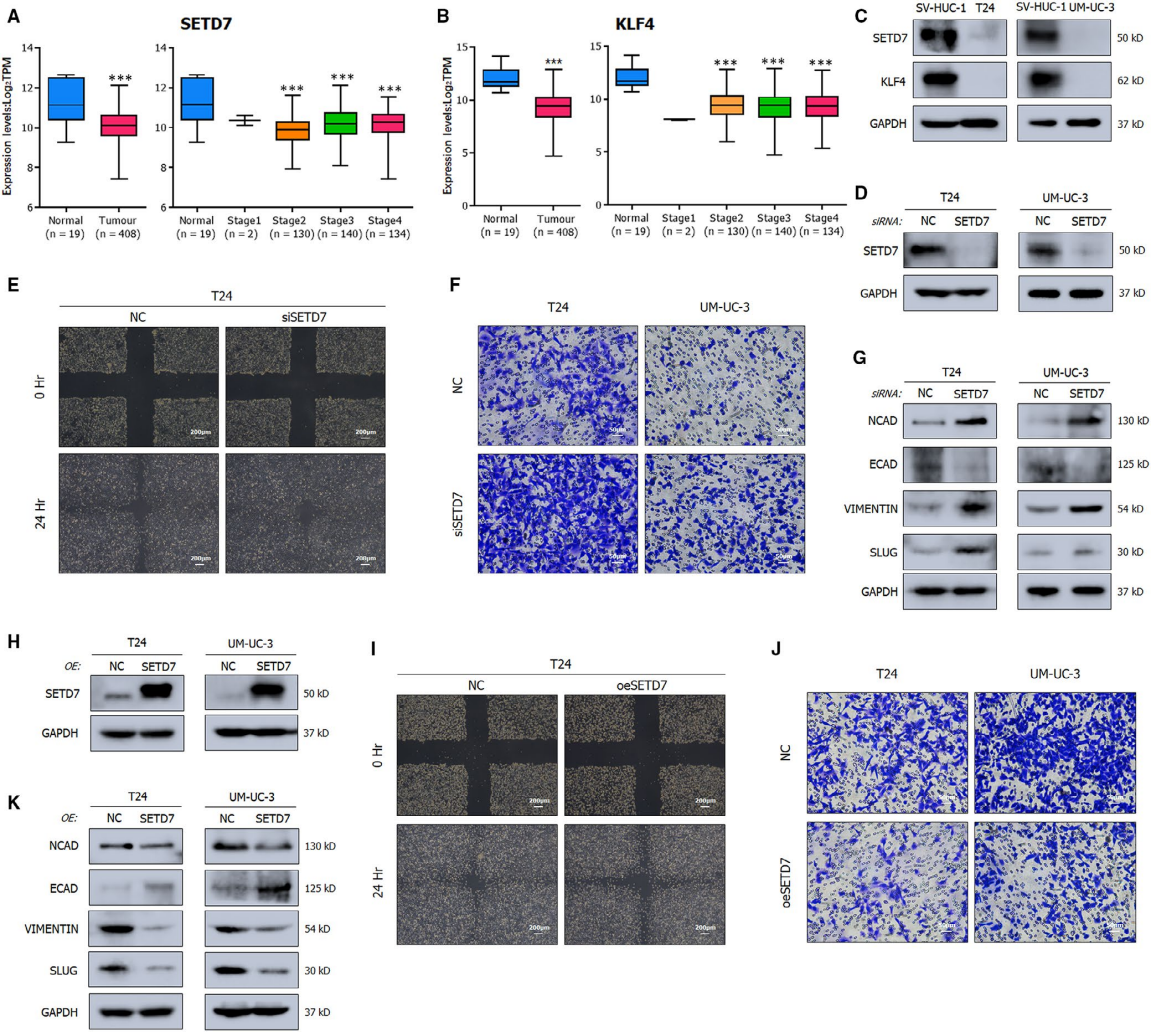
SETD7 and KLF4 act as tumour suppressors in BCa. A, B, Transcript levels of SETD7 and KLF4 in primary tumours in relative to normal subjects and mRNA expression levels among stages from TCGA database. C, Representative Western blots demonstrating the nearly deleted expression levels of SETD7 and KLF4 in BCa cell lines (T24, UM‐UC‐3) compared to the human normal urothelium cell line (SV‐HUC‐1). GAPDH was used for normalization. D, Western blot assay showing the deletion of SETD7 by the siRNA pool in BCa cell lines. E, F, Wound healing assay (E) and trans‐well assay (F) showing the promoted migration upon SETD7 depletion in cancer cell lines. G, Representative Western blots showing the altered EMT‐associated proteins upon the deleted SETD7. GAPDH served as normalization control. H, Western blot assay showing the overexpressed SETD7 by plasmids. I, J, Wound healing assay (I) and trans‐well assay (J) showing that overexpressed SETD7 inhibits the migration of cancer cell lines. K, Representative Western blots describing the inhibited migration with EMT‐associated proteins. Statistical significance was determined by Student's *t* test: ***P < .001

As observed in the analysis of the TCGA database, the expression of SETD7 and KLF4 was repressed in BCa compared to normal samples (Figure 6A,B). The decreased expression was also observed in distinct cancerous cell lines (Figure 6C). Our previous studies have demonstrated the explicit anti‐cancer role of KLF4 in BCa,^24^ which has also been acknowledged in other urologic cancers, such as prostate cancer^26^ and renal cancer.^27^ Therefore, we focused on exploring the mechanism of SETD7. Monitored by wound healing assay (Figure 6E) and trans‐well assay (Figure 6F), deletion of SETD7 using the homolog‐specific siRNA pool resulted in decreased migration activity, while overexpression of SETD7 via plasmids presented the opposing results (Figure 6I,J). Moreover, the tumour metastasis related genes whose levels were substantially affected by SETD7 were consistent with the roles of METTL3 and YTHDF2 in EMT progression (Figure 6G,K). The phenomenon further highlighted the interaction between SETD7 and METTL3/YTHDF2.

### 3.6 M^6^A/SETD7/KLF4 axis is established in regulating BCa progression

**Figure 7:**
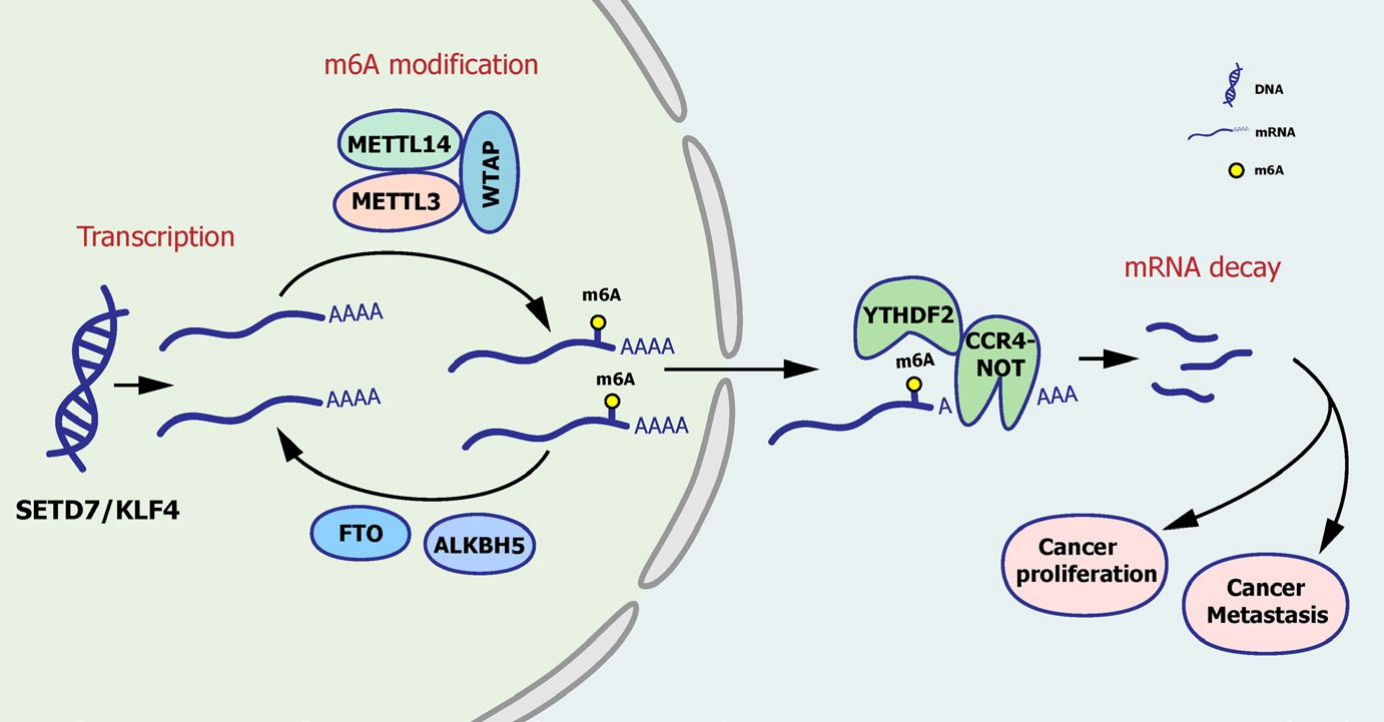
M^6^A/STED7/KLF4 axis is established in regulating BCa progression. In BCa, m^6^A modification is assembled on mRNA by METTL3 with other accessory catalytic subunits and eliminated by FTO and ALKBH5. YTHDF2 specifically recognizes m^6^A‐mRNA and induces the degradation of target mRNAs, promoting tumorigenesis eventually

Collectively, these data emphasized the importance of SETD7 and KLF4 mRNA degradation by the METTL3/YTHDF2 m^6^A axis in the proliferation and metastasis of BCa. The regulatory network of m^6^A modification is depicted in Figure 7.^11^

## 4 DISCUSSION

m^6^A modification is widely accepted as the most prevalent internal modification of mRNA in mammalian cells. The key components, responsible for the modification mechanism as ‘writer’, ‘eraser’ and reader, have been illuminated gradually with advances in research. Due to the rapid development of detection techniques, RNA modifications, which are as important as the modifications of DNA and protein, attain an increasing attention for their participation in physiological and pathological processes, such as embryo growth and development, human circadian rhythm,^28^ various diseases and multiple types of cancer.

In these studies, m^6^A modification is implicated in tumorigenesis, which is the core issue of concerned. Currently, m^6^A modification has been found to be associated with leukaemia,^29^ liver cancer,^30^ brain tumour,^31^ breast cancer^32^ and lung cancer.^16^, ^33^ For a better interpretation of molecular mechanisms, a complete modification process mediated by the cooperation of m^6^A writers, erasers and readers, rather than a single isolated gene, is needed. For instance, METTL3 was documented to degrade SOCS2 mRNA to promote the progression of liver cancer in a YTHDF2‐mediated m^6^A‐dependent manner.^23^ Methyltransferase METTL14, demethylase ALKBH5 and reader protein YTHDF3 were discovered to alter m^6^A levels in breast cancer, influencing the cell cycle, epithelial‐mesenchymal transition (EMT) and angiogenesis of cancer cells.^22^ Moreover, m^6^A‐increased translation of SNAIL mediated by YTHDF1 was confirmed as a part of EMT altered by disorder of METTL3 in cancer cell.^34^ However, few studies connect m^6^A modification with BCa, especially involving mutual collaboration between these m^6^A regulators.

Here, we demonstrated that disturbances in the balance of m^6^A modification mediated by the METTL3/YTDHF2 axis played a crucial role in tumour cell proliferation and migration thus influencing the tumorigenesis of BCa. When the expression of METTL3 was repressed, cells were primarily arrested in G1 phase and hardly proliferated or migrated in vitro; these cells had decreased m^6^A levels in total RNA. In vivo, concordantly, the depleted expression of METTL3 triggered the inhibition of tumorigenesis and metastasis. Considering the identified modification process^11^ and a previous study on liver cancer,^23^ we showed that cells with down‐regulated YTHDF2 were deficient in mRNA clearance capabilities and had impeded migration, which was in accordance with some of the functional roles of METTL3. Consequently, we explained that YTHDF2 recognized the m^6^A modification catalysed by METTL3 and completed the regulation of downstream targets.

In our transcriptome sequencing and MeRIP‐RT‐qPCR confirmation, SETD7 and KLF4 were perceived as the critical downstream targets of METTL3 and were indeed regulated in an m^6^A‐dependent manner. The negative correlation between targets and METTL3 reminded us of mRNA degradation mediated by the m^6^A reader YTHDF2. Interestingly, our data showed direct binding between YTHDF2 and the mRNA of targets. Therefore, we concluded that a METTL3/YTHDF2‐SETD7/KLF4 m^6^A axis exists in BCa. Herein, we investigated the dysregulation of SETD7 and KLF4 was partially inhibited by m^6^A modification, which was consistent with the KLF4‐m^6^A regulation in previous studies.^35^

Actually, the roles of SETD7 and KLF4 in cancers are controversial. As a member of histone lysine methyltransferase, SETD7 is also an important modifier of non‐histone proteins. While SETD7 probably drives the progression of gastric cancer,^36^ the modification of SETD7 would favour the tumour suppressor function of E2F1 in breast cancer.^37^ The important transcription factor KLF4, acted as a suppressor in metastasis and proliferation of cancer cells in colorectal cancer,^38^ while driven expression of KLF4 in tumour‐associated macrophages promoted immunosuppression to facilitate tumour growth in glioblastoma.^39^ However, by means of this study and our previous research,^24^ we demonstrated that SETD7 and KLF4 were two evident tumour suppressor genes in BCa. Consequently, the degradation of tumour suppressor genes by the m^6^A regulatory axis probably plays a vital role in the tumorigenesis of BCa.

In summary, we make a compelling illustration that the overexpressed METTL3 in BCa, the major methyltransferase catalysing m^6^A modification of mRNA, disrupts the dynamic balance of m^6^A modification, which is perceived as one of the vital reasons for cancer proliferation and cancer metastasis. In the m^6^A modification mechanism, our studies emphasize that the METTL3/YTHDF2‐SETD7/KLF4 axis plays an indispensable part. This m^6^A regulatory axis contains different types of modification, especially mRNA and protein, revealing a sophisticated network in the regulation of gene expression. Prospectively, these findings may supplement the underlying molecular mechanism of gene regulation in the tumorigenesis of BCa and provide a promising strategy for BCa therapy in the future.

## 5 CONCLUSIONS

Altogether, our study demonstrated a complete and important m6A regulatory pathway in BCa, containing the core ‘writer’ protein METTL3 and the major reader protein YTHDF2. The m^6^A modification catalysed by METTL3 is recognized by YTHDF2 to mediate the mRNA decay of tumour suppressors SETD7 and KLF4, which consequently induces the BCa progression. The METTL3/YTHDF2‐SETD7/KLF4 m^6^A axis is an intricate network of multiple modifications and could be a potential target for the diagnosis and treatment of BCa.

## CONFLICT OF INTEREST

The authors declare that there are no conflicts of interest in connection with the work submitted.

## AUTHORS' CONTRIBUTIONS

LX, XW and XZ proposed the conception of the study and designed the experiments; LX, XW, XX and BL revised the manuscript; HX, JL and YY performed the experiments, analysed the data and drafted the initial manuscript; HY and KJ contributed to animal experiments; XM and LH analysed the data and organized the figures; all authors read and approved the final manuscript.

## Data Availability

All data generated or analysed during this study are included in this published article.
