# Supplementary figures and images for: METTL3/YTHDF2 m6A axis promotes tumorigenesis by degrading SETD7 and KLF4 mRNAs in bladder cancer
Source: J Cell Mol Med. 2020 Mar 3;24(7):4092–104. doi: 10.1111/jcmm.15063 (PMC7171394; doi:10.1111/jcmm.15063)

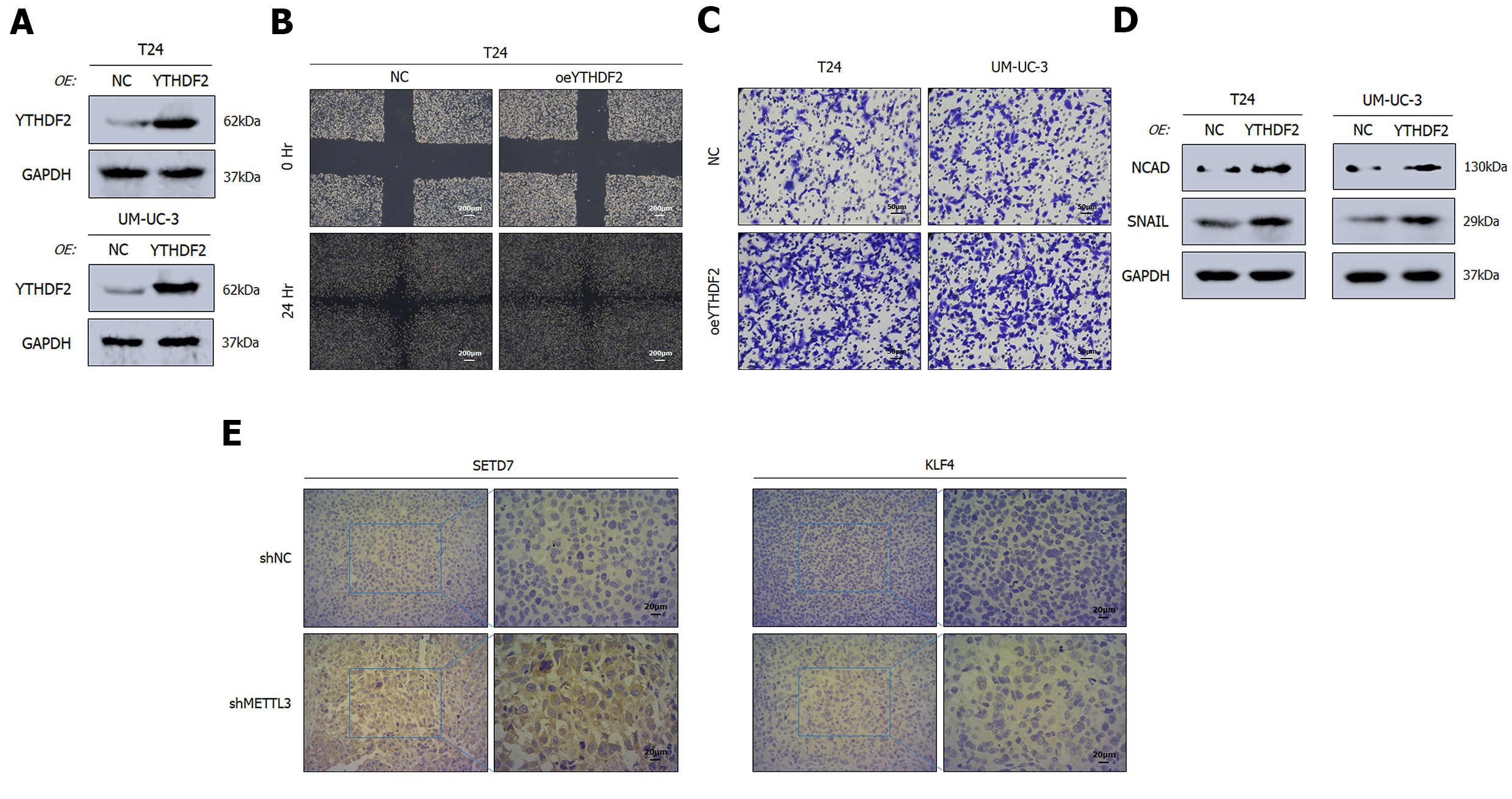

Supplement: Supplementary file 1 [file JCMM-24-4092-s001.tif]

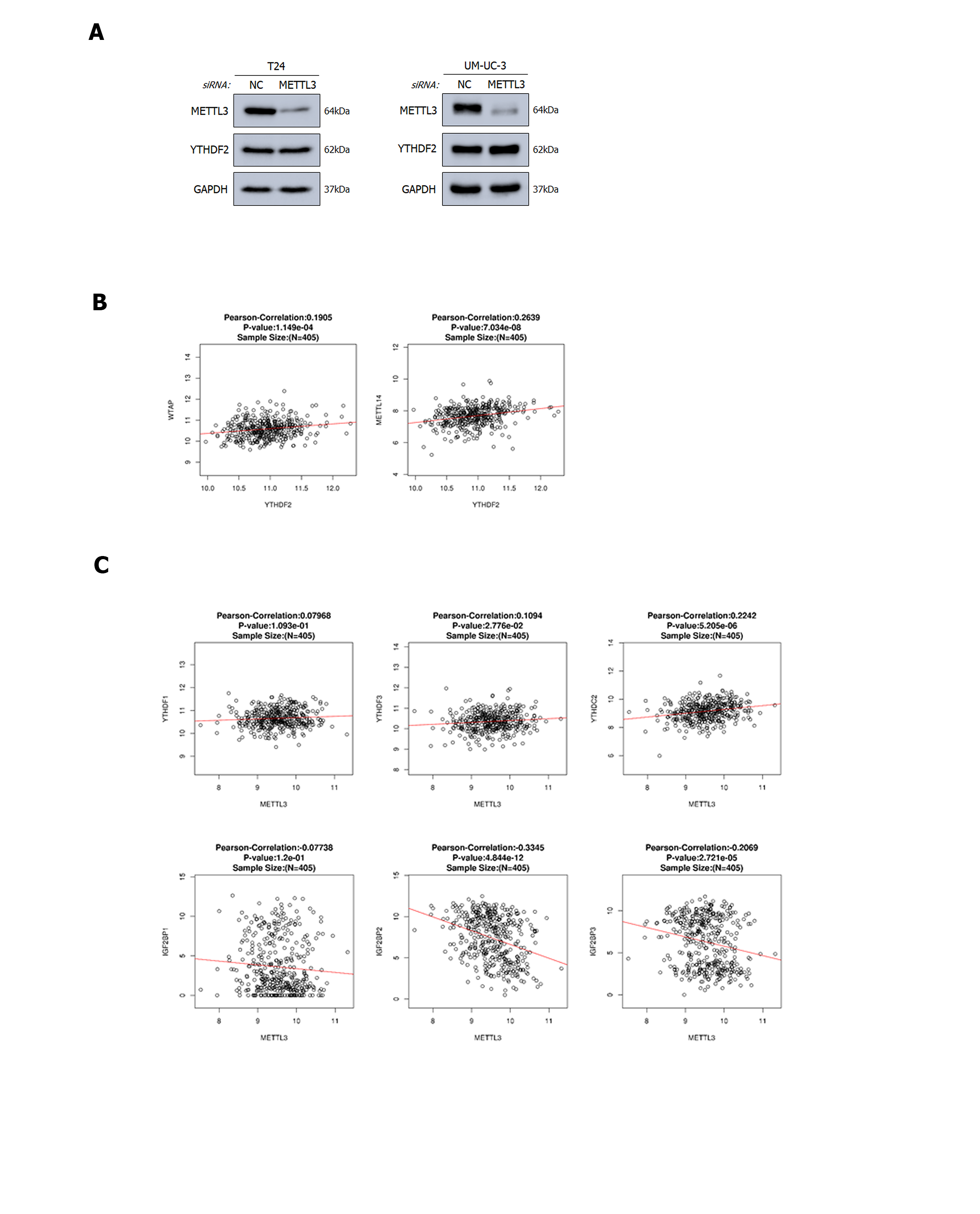

Supplement: Supplementary file 2 [file JCMM-24-4092-s002.tif]

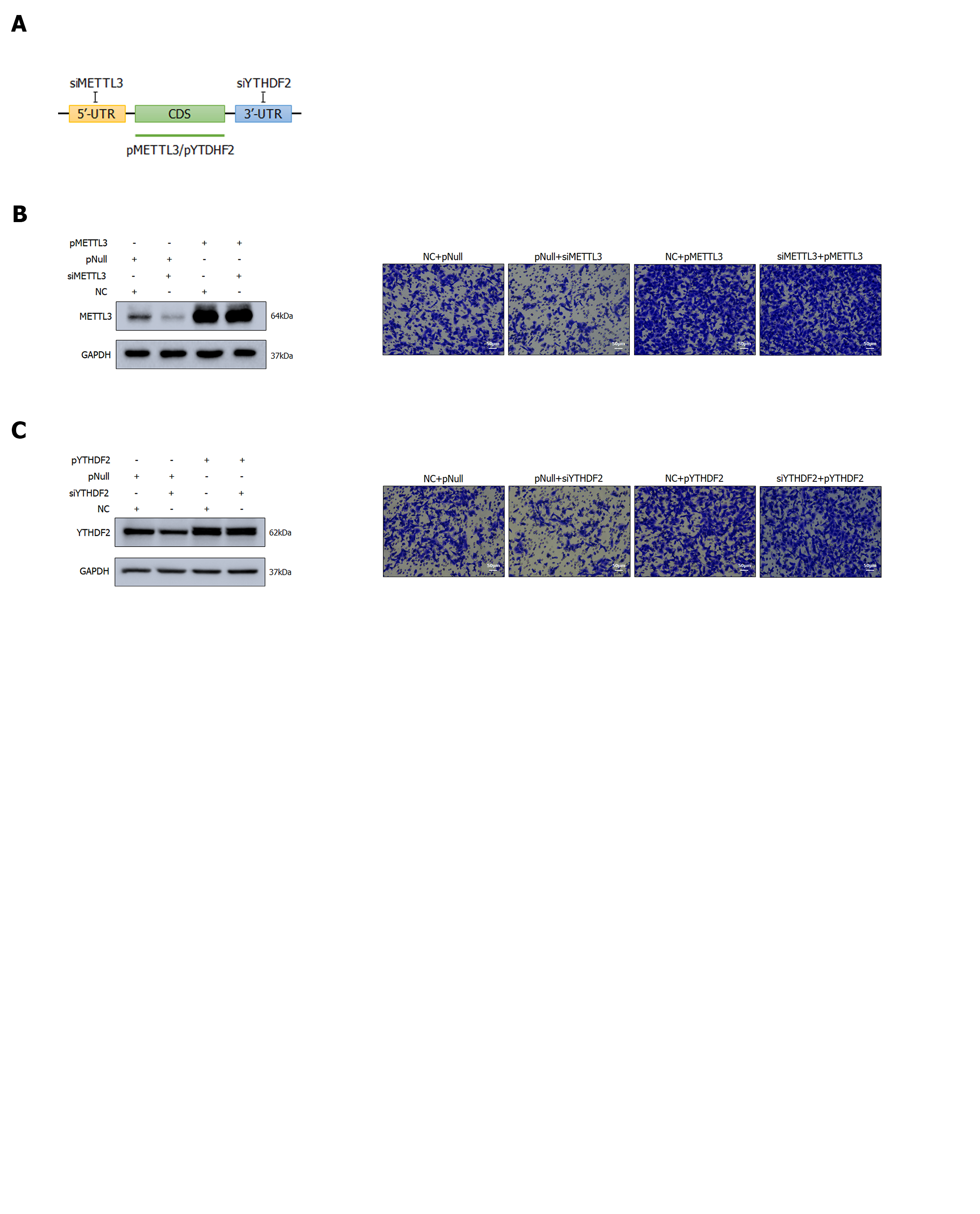

Supplement: Supplementary file 3 [file JCMM-24-4092-s003.tif]

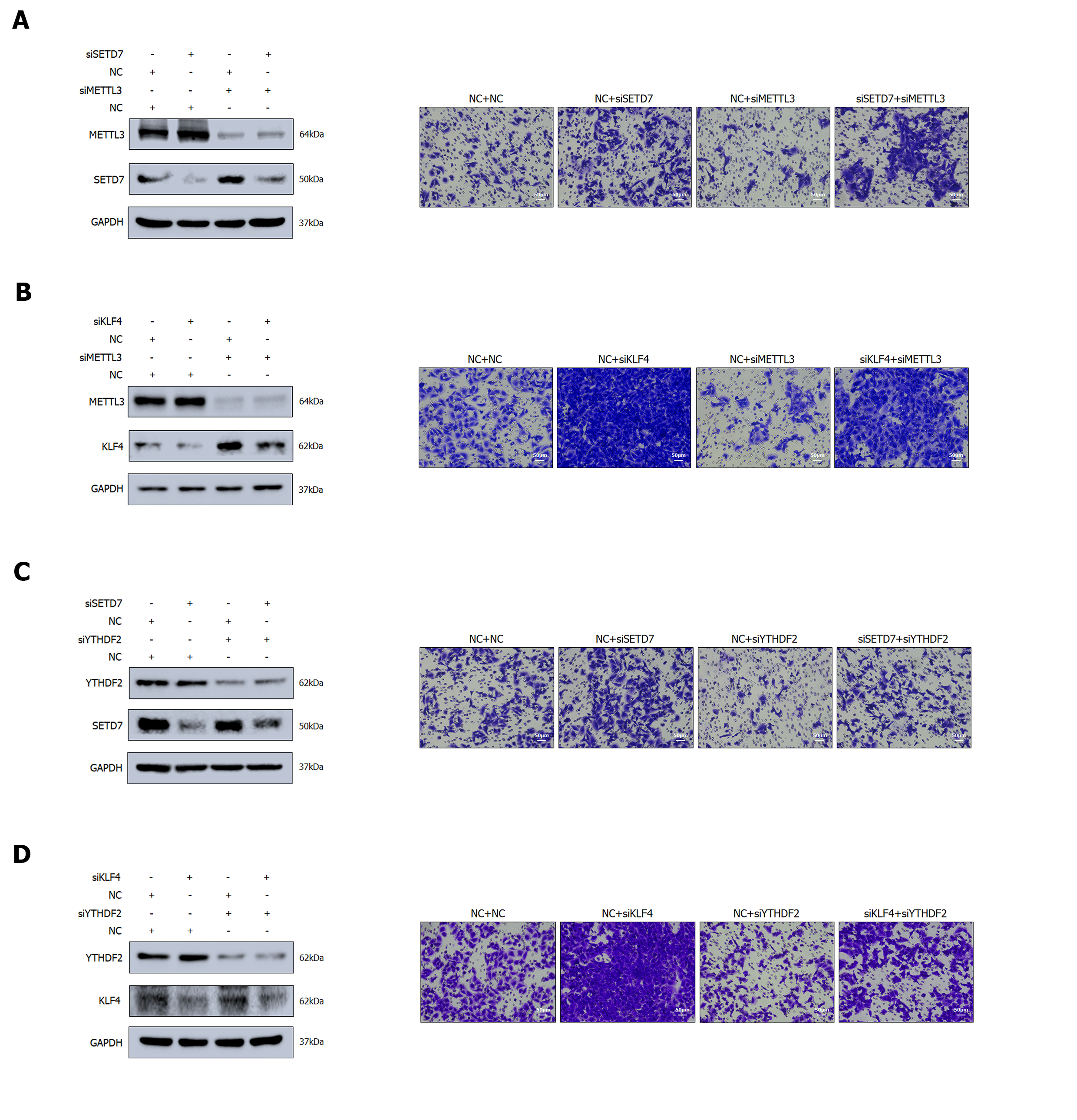

Supplement: Supplementary file 4 [file JCMM-24-4092-s004.tif]
