# Supplementary material for: METTL3/YTHDF2 m6A axis promotes tumorigenesis by degrading SETD7 and KLF4 mRNAs in bladder cancer
Source: J Cell Mol Med. 2020 Mar 3;24(7):4092–104. doi: 10.1111/jcmm.15063 (PMC7171394; doi:10.1111/jcmm.15063)
Supplement: Supplementary file 5 [file JCMM-24-4092-s005.docx]

**Supplementary Figure S1.** A. Representative western blots reveal the overexpressed YTHDF2 by plasmids in BCa cell lines. B-C. Wound healing assay (B) and trans-well assay (C) demonstrating the overexpressed YTHDF2 promotes cancer cell lines to migrate. D. Western blot assay documenting the upregulated expression level of proteins that promote metastasis and GAPDH served as the normalization control. E. Representative immunohistochemistry results show the evaluated expression of downstream targets SETD7 and KLF4 in UM-UC-3 derived xenograft tumor tissue.

**Supplementary Figure S2.** A. Representative western blots reveal the unchanged expression of YTHDF2 upon METTL3 depletion in BCa cell lines. B. Pearson correlation analysis between YTHDF2 and METTL14 or WTAP in the TCGA database by LinkedOmics online analysis website. C. Pearson correlation analysis between METTL3 and several m^6^A reader proteins.

**Supplementary Figure S3.** A. A schematic diagram states the location targeted by siMETTL3 or siYTHDF2 and siRNA-resistant exogenous METTL3 or YTHDF2. B. Rescue experiments showed the repressed migration ability of T24 cell line by knocking down METTL3 was eventually rescued by pMETTL3. C. Rescue experiments showed the repressed migration ability of T24 cell line by knocking down YTHDF2 was abrogated by pYTHDF2.

**Supplementary Figure S4.** A. Rescue experiments showed the repressed migration ability of T24 cell line by depletion of METTL3 was rescued by silencing of SETD7. B. Rescue experiments showed the repressed migration ability of T24 cell line by depletion of METTL3 was rescued by silencing of KLF4. C. Rescue experiments suggested the suppressed migration ability of T24 cell line by knocking down YTHDF2 was rescued by silencing of SETD7. D. Rescue experiments suggested the suppressed migration ability of T24 cell line by knocking down YTHDF2 was rescued by silencing of KLF4.
